# Digital quantification of stroma percentage enhances prognostic stratification in pancreatic cancer

**DOI:** 10.1016/j.sopen.2026.01.002

**Published:** 2026-01-19

**Authors:** Axel Bengtsson, Roland Andersson, Bodil Andersson, Daniel Ansari

**Affiliations:** Department of Surgery, Clinical Sciences Lund, Lund University, Skåne University Hospital, Lund, Sweden

**Keywords:** Pancreatic cancer, Tumor stroma percentage, Digital analysis, Prognosis

## Abstract

**Background:**

Pancreatic ductal adenocarcinoma (PDAC) is characterized by a prominent desmoplastic stroma, which plays a crucial role in tumor biology and treatment resistance. While the stromal compartment is a defining histopathological feature of PDAC, its prognostic significance remains incompletely understood. This study aimed to quantify the stromal content in PDAC using digital pathology and evaluate its association with patient outcomes.

**Methods:**

Tissue microarrays (TMAs) were constructed from resected PDAC specimens (*n* = 142). Digital analysis of tumor stroma percentage (TSP) was performed on tissue sections labeled with CA19–9. Cases were stratified into low and high TSP groups based on an optimized threshold of 44.2%. Associations between TSP and clinicopathological variables were assessed, and survival outcomes were analyzed using Kaplan-Meier and Cox proportional hazards models.

**Results:**

Digital quantification revealed wide intertumoral variability in TSP. A total of 127 (89%) patients were categorized into the high TSP group (>44.2% stroma). A high TSP was significantly associated with anatomic location of the tumor in the head of the pancreas. Patients with high TSP exhibited significantly prolonged overall survival (median: 27.8 months vs 12 months, *p* < 0.001). In multivariable analysis, high TSP remained an independent predictor of favorable prognosis (HR = 0.26, 95% CI: 0.13–0.52, *p* < 0.001).

**Conclusion:**

A high TSP is independently associated with improved survival in PDAC. These findings challenge traditional views of the stroma as purely tumor-promoting and suggest a potential protective role of the stromal compartment in certain contexts.

## Introduction

Pancreatic ductal adenocarcinoma (PDAC) has the highest mortality rate among all major solid cancers, with most tumors detected at an advanced stage, with limited oncotherapeutic advancements [1]. Despite nearly 500 phase 1 clinical trials, and 85 phase III clinical trials since the beginning of the century, advanced pancreatic cancer median survival is still less than 1 year. During this time, five new FDA-approved drugs have been introduced, showing little to no actual advantage for patient survival or quality of life [2].

A hallmark of PDAC is its extensive desmoplastic stroma. The stroma includes cancer-associated fibroblasts (CAFs), immune cells, endothelial cells, neural elements and extracellular matrix (ECM) proteins, such as collagen, fibronectin, and hyaluronic acid [3]. It has a complex and dual role in PDAC with both tumor-restraining and tumor-promoting properties [4]. On one hand, the dense fibrotic stroma encapsulates the tumor, physically limiting early spread and acting as a barrier to invasion [5], [6]. On the other hand, the stroma creates a protective niche that can promote tumor survival by creating an immunosuppressive environment, which together with poor vascularization and impaired drug delivery, contributes to tumor aggressiveness and treatment resistance [7], [8], [9].

The prognostic role of tumor stroma in PDAC is influenced not only by its amount, but also by its structural characteristics. Quantitatively, a larger or denser stroma generally predicts better outcomes, hinting at a tumor-restraining, physical containment role [10], [11], [12], [13], [14], [15]. Qualitatively, stroma subtype matters; mature, collagen-rich stroma is protective, whereas immature or activated stroma, high in α-SMA-positive CAFs, signals aggressiveness [16], [17]. Thus, histopathological evaluation of PDAC stroma may provide valuable prognostic information and aid in patient risk stratification.

The shift from traditional pathology to digital pathology has enabled more effective and objective histopathological evaluation of tumor specimens, including stromal assessments [18]. However, despite these advancements, there is still no consensus on the optimal threshold, methodology, or clinical utility of tumor stroma in PDAC.

The aim of this study was to evaluate the tumor stroma in PDAC and to determine its association with clinicopathological variables and patient outcomes using TMA-based sampling and digital pathology tools. By focusing on the tumor stroma as a single entity, without subdivision into smaller elements, we sought to develop a standardized and simplified approach that could be of value for clinical application.

## Materials and methods

### Data source

The study cohort consisted of consecutive patients with histopathologically confirmed PDAC undergoing pancreatic resection with curative intent between 1995 and 2017 at Skåne University Hospital Lund and Malmö, Sweden, with available formalin-fixed, paraffin-embedded tumor tissue. The cohort has been previously described [19]. Clinical, histopathological, and treatment data were obtained from pathology and hospital records. Staging was performed according to the eighth edition of the AJCC staging system.

### Immunohistochemical staining

Tissue microarrays (TMAs) were constructed as previously described [19]. In brief, areas representing cancer were marked on hematoxylin/eosin-stained sections. Four 2 mm tissue cores were then extracted and mounted using an automated tissue array device (Minicore® 3, Alphelys, Plaisir, France). For immunohistochemical analysis, 3 μm TMA-sections were automatically pre-treated using the PT-link system (Dako, Agilent Technologies, Glostrup, Denmark). The individual TMA-slides were incubated with the CA19-9 primary antibody (ab289665, dilution 1:500, Abcam, Amsterdam, Netherlands). Slides were then washed and stained with a biotinylated anti-mouse secondary antibody (BA-2000, dilution 1:200, Vector Laboratories, Burlingame, CA, USA). For signal amplification, avidin-biotin-peroxidase complex (Vectastain Elite ABC-HRP Kit, Vector Laboratories, Burlingame, CA) was utilized and antibody-antigen complexes were visualized with chromogen diaminobenzidine (DAB) (Vector Laboratories).

### Imaging and stroma quantification

Immunolabeled TMA slides were scanned using a Hamamatsu S210 microscope slide scanner (Hamamatsu, Japan). The TMA tissue to be analyzed was annotated using the NDPview software (Hamamatsu, Japan). Artefacts, such as folds, necrotic or other non-relevant regions were excluded from the sections. A Python script was used to extract the x and y coordinates of the region of interest (ROI). An ImageJ script was then used to extract each TMA ROI as a jpg file. An ImageJ script for color deconvolution was used to separate each image into two images, one immunolabeled (DAB-brown) and one hematoxylin (blue) stained image. The DAB labeling intensity was determined by using three thresholds; low, medium and high (0 to 3) intensity and the distribution of labeling of the total area (0–100%) was recorded. The tumor stroma percentage (TSP) was digitally analyzed via ImageJ using a combination of threshold, Gaussian blur and particle size to select the significant DAB staining. The selected area was cleared from the image leaving the stroma part of the tissue. The area of the stroma was divided by the total tissue area to get % DAB in stroma. Some tissue sections could not be digitally analyzed for stromal area, due to poor separation between labeled and non-labeled structures. These sections were manually assessed by two analysts to estimate the stromal area. For each patient, the TSP was calculated using the average of up to four distinct tumor areas.

### Statistical analysis

Clinicopathological variables were compared using the Mann-Whitney *U* test for continuous variables and the chi-square test for categorical variables, with Fisher's exact test applied when expected counts were below five. Survival estimates were calculated by the Kaplan Meier method and survival differences between groups were assessed by the log-rank test. Cox proportional hazards models were used for estimation of hazard ratios (HR) for overall survival according to TSP in uni- and multivariable analysis adjusting for age, gender, ASA score, tumor location, AJCC stage, tumor differentiation, resection margin status and adjuvant chemotherapy. To determine the optimal cut-off for TSP for survival analysis, maximally selected rank statistics from the ‘maxstat’ R package were used. The optimal stroma percentage cut-off value was determined to be 44.2%. To evaluate whether the cut-off identified by maxstat was supported by the continuous relationship between TSP and survival, we performed a spline-based sensitivity analysis. Restricted cubic splines (RCS) with three knots were incorporated into a multivariable Cox proportional hazards regression model, using TSP as a continuous predictor. Knots were placed at default quantiles of the distribution. The model was adjusted for the same covariates used in the primary multivariable analysis. All statistical analyses were performed using Stata/MP (version 18.0) and RStudio (version 2025.09.2).

## Results

Clinicopathological characteristics of the patients are presented in Table 1. The median age was 69 years and 49% were female. Most tumors were located in the head of the pancreas (85%) and the majority (76%) received adjuvant chemotherapy. No patient received neoadjuvant treatment. The median survival was 24.1 months, and the 5-year survival rate was 16%.

**Table 1:** Patient characteristics.

Variable	TSP low (*n* = 15)	TSP high (*n* = 127)	*P*-value
Age	72 (67–73)	68 (63–73)	0.166
Female	7 (46.7%)	62 (48.9%)	0.875
ASA score			0.308
I	0 (0%)	12 (9.4%)	
II	7 (46.7%)	71 (55.9%)	
III	7 (46.7%)	40 (31.5%)	
Missing	1 (6.7%)	4 (3.2%)	
Tumor location (head)	10 (66.7%)	110 (86.7%)	0.043
Tumor size (cm)	3.5 (2.5–4.0)	3.0 (2.5–4.0)	0.378
AJCC Stage			0.381
I	3 (20.0%)	21 (16.5%)	
II	8 (53.3%)	54 (42.5%)	
III	3 (20.0%)	51 (40.2%)	
Missing	1 (6.7%)	1 (0.8%)	
Tumor differentiation			0.241
Well-moderate	4 (26.7%)	53 (41.7%)	
Poor	11 (73.3%)	72 (56.7%)	
Missing	0 (0%)	2 (1.6%)	
Resection margin status (positive)	3 (20.0%)	53 (42.1%)	0.099
Adjuvant chemotherapy			1.000
None	2 (15.4%)	19 (16.4%)	
Received	11 (84.6%)	97 (83.6%)	
Missing	2 (13.3%)	11 (8.7%)	

AJCC, American Joint Committee on Cancer; ASA, American Society of Anesthesiologists.

### Stroma assessment

**Fig. 1:**
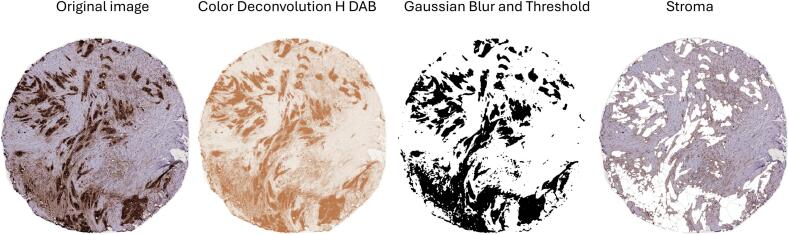
Computer aided quantification of TSP in a CA19-9 labeled pancreatic cancer TMA core.

**Fig. 2:**
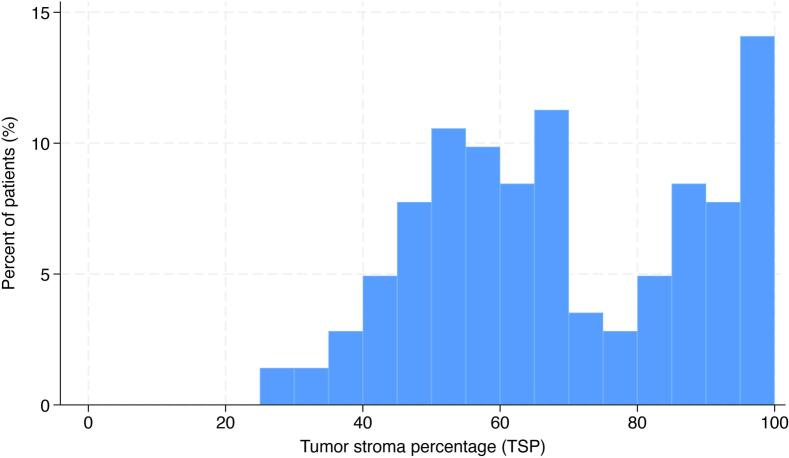
Histogram showing the distribution of TSP among the pancreatic cancer patients.

The tumor stroma was evaluated after digital quantification and optimization. Different values for threshold, Gaussian blur and particle size, were tested. This resulted in division of the sections that were “easy” (good contrast between labeled tumor cells and stroma) or “difficult” (poor contrast) to evaluate. Some “difficult” sections were visually evaluated (10%). Finally, the same threshold value were used for all remaining sections, but with two different combinations of values for Gaussian blur and particle size (Fig. 1). The distribution of TSP among patients is shown in Fig. 2. The median TSP was 66.1%, ranging from 27.8% to 99.9%. In total, 127 (89%) patients were classified as having high TSP (>44.2%), while 15 (11%) patients were classified as having low TSP (≤44.2%).

### Correlation of TSP with clinicopathological parameters

A high TSP was significantly associated with anatomic location of the tumor (pancreatic head), 87% vs 67% (*p* = 0.043). TSP was not associated with age, gender, ASA score, tumor size, stage, grade, margin status or receipt of adjuvant treatment (Table 1).

### Survival analysis

**Fig. 3:**
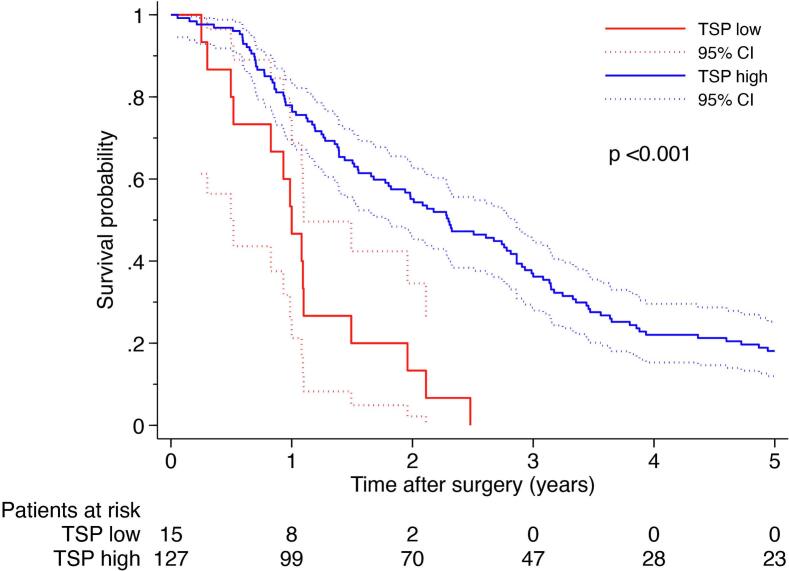
Overall survival of patients with resected pancreatic cancer stratified by TSP based on the maxstat cut-off.

Kaplan-Meier survival curves demonstrated that a high TSP was significantly associated with prolonged overall survival (median 27.8 months vs 12 months, *p* < 0.001), Fig. 3. The 5-year survival was 18% in the high-TSP group vs 0% in the low-TSP group. A high TSP was associated with improved survival in univariable (HR = 0.28, 95% CI 0.16–0.49, *p* < 0.001) and multivariable Cox regression models adjusting for age and gender (HR = 0.27, 95% CI 0.15–0.48, p < 0.001), as well as additional clinical variables (HR = 0.26, 95% CI: 0.13–0.52, p < 0.001), Table 2.

**Table 2:** Multivariable Cox regression analysis.

Variables	HR	95% CI	P-value
Unadjusted			
TSP high	0.28	0.16–0.49	<0.001
Adjusted for age and gender			
TSP high	0.27	0.15–0.48	<0.001
Adjusted for 8 covariates^a^			
TSP high	0.26	0.13–0.52	<0.001

a Adjusted for age, gender, ASA score, tumor location, AJCC stage, tumor differentiation, resection margin status and adjuvant chemotherapy.

**Fig. 4:**
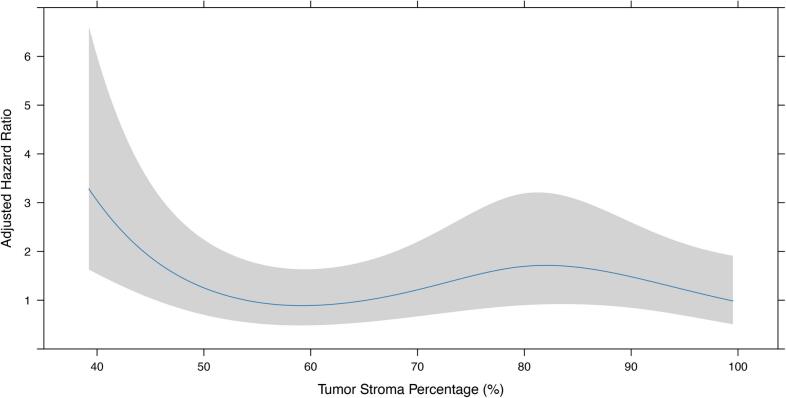
Restricted cubic spline showing the adjusted hazard ratio (HR) for overall survival across the continuous distribution of TSP.

As a sensitivity analysis to validate the cut-off identified using maximally selected rank statistics (maxstat), we modelled TSP as a continuous variable using restricted cubic splines within an adjusted Cox regression framework. The spline analysis demonstrated a non-linear association between TSP and survival (Fig. 4). A statistically significant elevation in risk (HR > 1 with 95% CI not crossing 1) was confined to a narrow interval at the lower end of the distribution. This region corresponded closely to the threshold identified by maxstat (44.2%), supporting the validity of this cut-off. These results confirm that tumors with “low stroma” (below ~45%) have the worst outcomes, consistent with the dichotomized analysis, where the “high stroma” group demonstrated markedly better prognosis.

## Discussion

In this study, we evaluated TSP using a reproducible digital image-analysis approach and demonstrated that stromal quantity is linked to patient outcomes in PDAC. Patients with a high TSP experienced significantly improved survival. These results highlight the prognostic utility of digitally derived stromal metrics and support the relevance of the stromal compartment in tumor biology.

Previous studies have shown conflicting results regarding the role of tumor stroma in PDAC. Early investigations predominantly highlighted the stroma's tumor-promoting attributes, including physical compression of vasculature, immunosuppression, and resistance to chemotherapy [22], [23], [24]. However, more recent studies have nuanced this view. For instance, it was observed that genetic or pharmacological depletion of stromal elements can lead to more aggressive tumor phenotypes and reduced survival in preclinical models [5], [25]. These findings have shifted the focus from stromal ablation to stromal modulation or reprogramming as a more viable therapeutic strategy.

Our data contribute to this evolving narrative by quantitatively assessing total stromal area and showing a significant association between high stromal content and improved survival outcomes. This aligns with previous findings, which reported that tumors with abundant stroma, particularly rich in mature collagen and fibroblastic content, were less likely to metastasize and were associated with better prognoses [13], [14], [26]. Furthermore, decreased stromal content has been observed in lymph node and solid-organ metastases compared to primary tumors [6], [26], suggesting that the stroma may act to confine tumor progression within the primary site, whereas stromal depletion might facilitate metastatic escape and growth in more permissive microenvironments.

Beyond volume, the biological characteristics of the stroma, defined by its cellular and acellular components, are instrumental in dictating its role in tumor progression. CAFs, immune infiltrates, vascular elements and ECM proteins, each contribute differently to tumor behavior. Subtypes of CAFs, including myofibroblastic CAFs (myCAFs), immunogenic CAFs (iCAFs), and antigen-presenting CAFs (apCAFs), have distinct effects on ECM production, immunosuppression, vascular remodeling and tumor progression [27], [28]. The spatial distribution of CAFs may also have important prognostic implications [29]. Although the bulk of ECM proteins are derived from stromal cells, tumor cells themselves also secrete ECM proteins. Tumor cell-derived ECM proteins appear to be pro-tumorigenic and associated with poor survival, while stroma-derived ECM proteins may either support or restrain tumor progression and correlate with either short or long survival [30]. Bulk and single-cell transcriptomics have categorized PDAC stroma into two major subtypes, including “activated” and “normal-like” subtypes [31], [32]. The “activated” stroma is rich in myCAFs, iCAFs, M2-like macrophages and regulatory T-cells, while the “normal-like” stroma supports pancreatic stellate cells, M1-like macrophages and effector/exhausted T-cells. These subtypes carry distinct prognoses, with activated stroma associated with worse outcomes, highlighting the importance of stromal phenotyping in addition to quantification.

These findings also have therapeutic implications. Clinical trials targeting the tumor stroma, such as those involving hedgehog pathway inhibitors or enzymatic degradation of hyaluronic acid, have largely yielded disappointing results, with some even accelerating disease progression [33], [34], [35]. These failures may be attributed to indiscriminate stromal depletion, which removes both protective and harmful elements. A more refined strategy involves stromal reprogramming, aimed at altering the functional phenotype of stromal components. Agents such as vitamin D analogs, TGF-β inhibitors or combination of FAK and RAF-MEK inhibition have shown promise in preclinical models by converting the stroma into a less tumor-promoting and more drug-permissive environment [36], [37], [38]. Several ongoing clinical trials are investigating strategies to modulate the tumor stroma by selectively targeting specific stromal components, such as CAFs, immune cells, ECM proteins and vasculature [39].

Limitations of our study include its retrospective nature and reliance on histological sections, which may not capture functional aspects or dynamic stromal changes over time. Additionally, our analysis was based on total stromal area, without distinguishing between specific cell types or ECM substructures, which could influence tumor behavior differently. Moreover, tumor heterogeneity and sampling bias remain potential confounders, particularly when using TMAs [40]. Variations in TMAs may occur due to the inherent heterogeneity of tumor tissues, technical challenges in sample collection and processing, and the quality and depth of the original donor blocks. These variations can include differences in tissue morphology, the absence or presence of certain molecular markers, and variations in the quality or quantity of tissue within each core, despite efforts to standardize analysis on a single slide.

Future directions include integrating digital pathology with multiplex imaging and spatial omics to better characterize the functional landscape of the stroma. Prospective studies correlating stromal features with treatment response and patient outcomes will be crucial to validate stromal metrics as prognostic or predictive biomarkers. In parallel, the development of therapies that selectively modulate stromal components, rather than nonselective ablation, holds promise for improving outcomes in PDAC.

In conclusion, our findings reinforce the notion that tumor stroma in pancreatic cancer is not uniformly tumor-promoting. Instead, a high stromal percentage may serve as a favorable prognostic marker and suggests that preserving or reprogramming the stroma, rather than ablating it, could offer therapeutic benefit. This underscores the need for nuanced approaches in targeting the tumor microenvironment.

## CRediT authorship contribution statement

**Axel Bengtsson:** Writing – original draft, Investigation, Formal analysis. **Roland Andersson:** Writing – review & editing, Supervision, Funding acquisition. **Bodil Andersson:** Writing – review & editing, Supervision. **Daniel Ansari:** Writing – review & editing, Visualization, Supervision, Methodology, Funding acquisition.

## Ethics approval

This study was approved by the Ethical Review Board at Lund University (Ref 2010/684, 2012/661, 2015/266, 2015/833, 2017/320) and the Swedish Ethical Review Authority (Ref 2022–02371-01 and 2025–07092-01). The study followed the STROBE [20] and REMARK [21] guidelines.

## Funding sources

This work was supported by the 10.13039/501100002794Swedish Cancer Society, the 10.13039/501100004359Swedish Research Council, the 10.13039/501100003173Crafoord Foundation, Regional Research Support and ALF funding from 10.13039/501100009780Region Skåne.

## Declaration of competing interest

There are no conflicts of interest to declare.
